# Functional and Taxonomic Traits of the Gut Microbiota in Type 1 Diabetes Children at the Onset: A Metaproteomic Study

**DOI:** 10.3390/ijms232415982

**Published:** 2022-12-15

**Authors:** Stefano Levi Mortera, Valeria Marzano, Pamela Vernocchi, Maria Cristina Matteoli, Valerio Guarrasi, Simone Gardini, Federica Del Chierico, Novella Rapini, Annalisa Deodati, Alessandra Fierabracci, Stefano Cianfarani, Lorenza Putignani

**Affiliations:** 1 Unit of Human Microbiome, Multimodal Laboratory Medicine Research Area, Bambino Gesù Children’s Hospital, IRCCS, 00165 Rome, Italy; 2 Diabetes & Growth Disorders Unit, Bambino Gesù Children’s Hospital, IRCCS, 00165 Rome, Italy; 3 GenomeUp SRL, 00165 Rome, Italy; 4 Infectivology and Clinical Trials Research Department, Bambino Gesù Children’s Hospital, IRCCS, 00165 Rome, Italy; 5 Department of Systems Medicine, University of “Tor Vergata”, 00133 Rome, Italy; 6 Department of Women’s and Children Health, Karolisnska Institute, University Hospital, 17177 Stockholm, Sweden; 7 Unit of Microbiology and Diagnostic Immunology, Bambino Gesù Children’s Hospital, IRCCS, 00165 Rome, Italy; 8 Unit of Human Microbiome, Department of Diagnostics and Laboratory Medicine, Bambino Gesù Children’s Hospital, IRCCS, 00165 Rome, Italy

**Keywords:** Type 1 diabetes, gut microbiota, metaproteomics, paediatric, insulin need, functional annotation, label-free quantitative analysis (LFQ)

## Abstract

Type 1 diabetes (T1D) is a chronic autoimmune metabolic disorder with onset in pediatric/adolescent age, characterized by insufficient insulin production, due to a progressive destruction of pancreatic β-cells. Evidence on the correlation between the human gut microbiota (GM) composition and T1D insurgence has been recently reported. In particular, 16S rRNA-based metagenomics has been intensively employed in the last decade in a number of investigations focused on GM representation in relation to a pre-disease state or to a response to clinical treatments. On the other hand, few works have been published using alternative functional omics, which is more suitable to provide a different interpretation of such a relationship. In this work, we pursued a comprehensive metaproteomic investigation on T1D children compared with a group of siblings (SIBL) and a reference control group (CTRL) composed of aged matched healthy subjects, with the aim of finding features in the T1D patients’ GM to be related with the onset of the disease. Modulated metaproteins were found either by comparing T1D with CTRL and SIBL or by stratifying T1D by insulin need (IN), as a proxy of β-cells damage, showing some functional and taxonomic traits of the GM, possibly related to the disease onset at different stages of severity.

## 1. Introduction

Type 1 diabetes (T1D) is a multifactorial disease characterized by a progressive destruction of pancreatic β cells, triggered by an autoimmune response, that leads to a lack of insulin production [1,2]. The insurgence of diagnostic T1D symptoms can occur at any age, but the incidence in children and young subjects is definitely higher; thus, it is nowadays considered one of the most common chronic pediatric disease [3,4]. After the hypothesis was formulated at the end of the eighties, gut microbial markers associated with insulin resistance and evidence of the role of gut microbiota (GM) in the immune-mediated onset of T1D were observed in rodent models [5,6,7,8]. Shifting to the human holobiont niche, where breeding conditions and environment cannot be controlled, as in an animal facility, some confounding factors must be taken into account, such as dietary habits and geographical location, to define how GM features could be related to the disease [9]. During the last decade, a decisive growth of studies on T1D related to human GM ensued in the publication of many papers and reviews about this topic [10,11,12,13,14,15]. Besides a small number of experiments based on PCR-denaturing gradient gel electrophoresis (PCR-DGGE) or real-time quantitative PCR (RT-qPCR), the majority of the investigation conducted so far used a 16S rRNA gene fragment metagenomic approach producing both consistent and contrasting evidence concerning specific microbial markers in the human GM [16,17,18,19]. The employment of the 16S rRNA metagenomics alone is not enough to provide a relevant functional insight into a complex microbial community such as the human GM. Other omic disciplines can be applied to describe the actual gene expression and modulation, and in this sense metaproteomics could indeed contribute to a more in-depth exploration of GM and its influence on human health and physiology [20]. Probably due to a higher number of challenging features, metaproteomic investigations are still much less frequently undertaken with respect to 16S rRNA-based metagenomics and, particularly about GM related to diabetes and obesity, only a few publications have been published so far [21]. Many important issues raised in metaproteomic practice have been managed or overcome in recent years. Moreover, a number of bioinformatics tools have been developed and are currently in use [22,23,24]. Nevertheless, comprehensive studies exploiting the complementarity of metaproteomics with respect to other meta-omics seem to be still suffering from the absence of a gold standard workflow for data management, from functional annotation to quantitative analysis of metaproteins. In particular, the taxonomic outcomes, given that meta-omics rely on analytical techniques working on different molecules, could result in much different results, especially when microbial populations are very complex and sample-specific databases (DB) are not available [25].

In this work, we applied a metaproteomic workflow to a cohort of T1D children to investigate the GM composition compared with that of some siblings (SIBLs) and aged matched controls (CTRLs). In particular, siblings were involved in the study to seek for shared familiar features of the GM to be related to the T1D onset, rather than to investigate common genetic traits that are already known to exist in T1Ds and their relatives [26,27,28]. It is reasonable that if particular aspects of the GM composition are recognized as a sort of microbial signature for T1D-affected individuals living in the same geographical areas, peculiar features could be observed as well in people sharing the same familiar environment and be considered as a therapeutic target for patients and for risky siblings [29].

Therefore, among the main aims of this work, to unveil the differential regulation of microbial proteins in T1Ds, compared with healthy CTRLs and SIBLs, was important to individuate those functions and molecular mechanisms involved in the onset of the disease or even in triggering the autoimmune response causing the insulin deficit.

To the best of our knowledge, only two case-control studies on T1D have been reported so far, using metaproteomics on fecal samples, particularly focusing on the connection between the gut metaproteome and host protein expression [30,31]. Integration of data was instead performed by Heintz-Buschart et al. in a multi-omic investigation on familial T1D, merging results from shotgun metagenomic, metatranscriptomic, and metaproteomic approaches [32]. Our results provided a functional rationale for some trends in bacterial distribution that have been already described in previous studies through metagenomic approaches and also highlighted GM features to be associated with the earlier stages of T1D onset.

## 2. Results

### 2.1. LC-MS/MS Analysis and GM Ecology

Analyses were performed on stool samples from 39 patients at onset (T1D), 15 siblings (SIBL), and 30 controls (CTRL), extracting 816.5 ± 322.6 µg (mean ± s.d.), 894.6 ± 253.4 µg, and 860.1 ± 285.2 µg of proteins, respectively. After tryptic digestion on 50 µg of total protein content, 35.79 ± 16.43 µg (mean ± s.d.) of peptides were recovered from T1D samples, 51.58 ± 15.14 µg from SIBL samples, and 37.69 ± 15.29 from CTRL samples (Table 1).

The LC-MS/MS analysis yielded 40,663 bacterial protein groups (PGs) overall and 512 human proteins with 216,926 unique peptide sequences. After the filtering steps, the list was reduced to 2175 bacterial PGs and 140 human proteins, with only two sequences shared between the two groups (Files S1 and S2).

**Figure 1:**
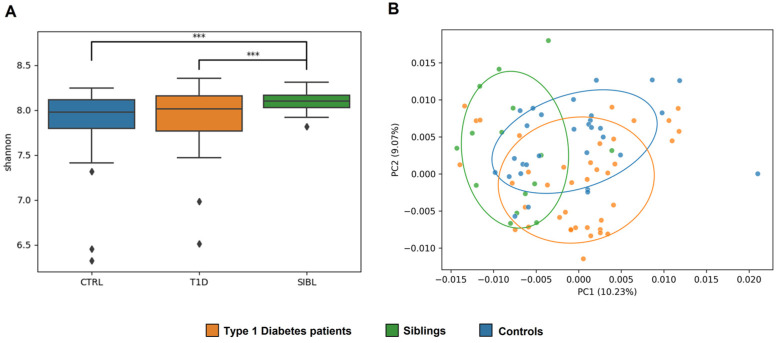
Analysis of GM ecology comparing the T1D, SIBL, and CTRL groups. **Panel** (**A**): Alpha diversity was calculated with QIIME using the Shannon diversity index and determining the *p*-value for group comparison by variance analysis (ANOVA). Significance (marked with *** when *p*-value < 0.01) was found for the comparisons between the T1D and SIBL groups (*p*-value = 0.009) and between CTRL and SIBL (*p*-value = 0.002). **Panel** (**B**): Principal coordinate analysis (PCoA) plots were constructed to illustrate the beta diversity of the samples, based on the Bray–Curtis distance matrix. To test the association between the covariates and beta diversity measures, a variance analysis method based on permutation was used (PERMANOVA, 9999 permutations, *p*-value = 0.0014).

The alpha diversity measure showed a statistically significant higher Shannon index for SIBLs compared with T1D (*p*-value = 0.009) and CTRL (*p*-value = 0.002), while the T1D and CTRL groups were more similar to each other (Figure 1A). Looking at beta diversity, a clear separation between the three groups was not observed overall, but again the SIBL group showed a higher distance from both T1D and CTRL (*p* value = 0.0014) (Figure 1B).

### 2.2. GM Quantitative Analysis: T1D vs. CTRL vs. SIBL

**Figure 2:**
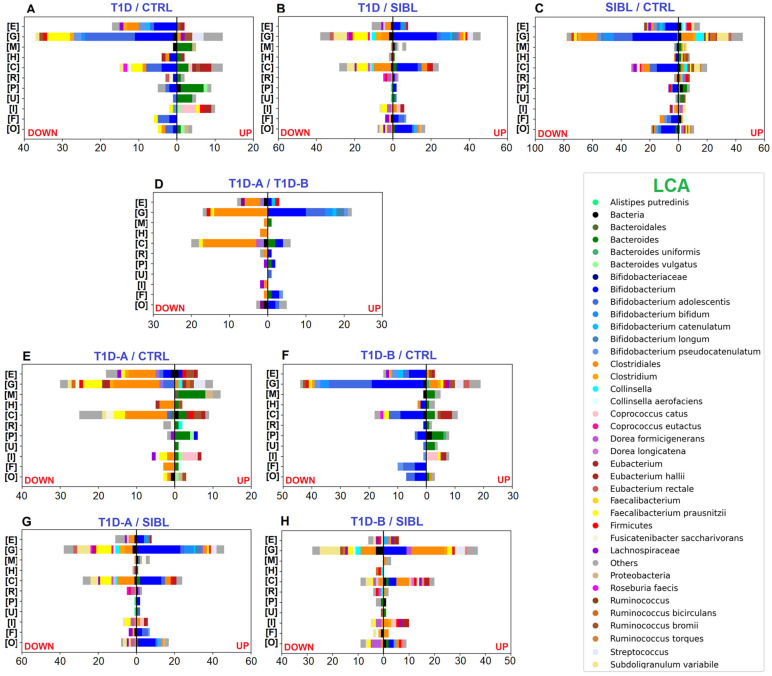
Bar charts plotting the number of statistically significant differential PGs, determined by the abundance ratio values in the comparison between groups of individuals and associated with functional annotation. Color code identifies taxa assigned to UP- (right) and DOWN-regulated (left) PGs by LCA. Selected COG categories: [C] Energy production and conversion—[E] Amino acid transport and metabolism—[F] Nucleotide transport and metabolism—[G] Carbohydrate transport and metabolism—[H] Coenzyme transport and metabolism—[I] Lipid transport and metabolism—[M] Cell wall/membrane/envelope biogenesis—[O] Posttranslational modification, protein turnover, chaperones—[P] Inorganic ion transport and metabolism—[R] General function prediction only—[U] Intracellular trafficking,secretion, and vesicular transport. **Panels** (**A**–**C**): comparisons between T1D, CTRL, and SIBL groups. **Panel** (**D**): comparison between groups of patients stratified by severity (T1D-A and T1D-B). **Panels** (**E**,**F**): tests comparing T1D-A and T1D-B groups versus CTRL. **Panels** (**G**,**H**): tests comparing T1D-A and T1D-B groups versus SIBL.

**Figure 3:**
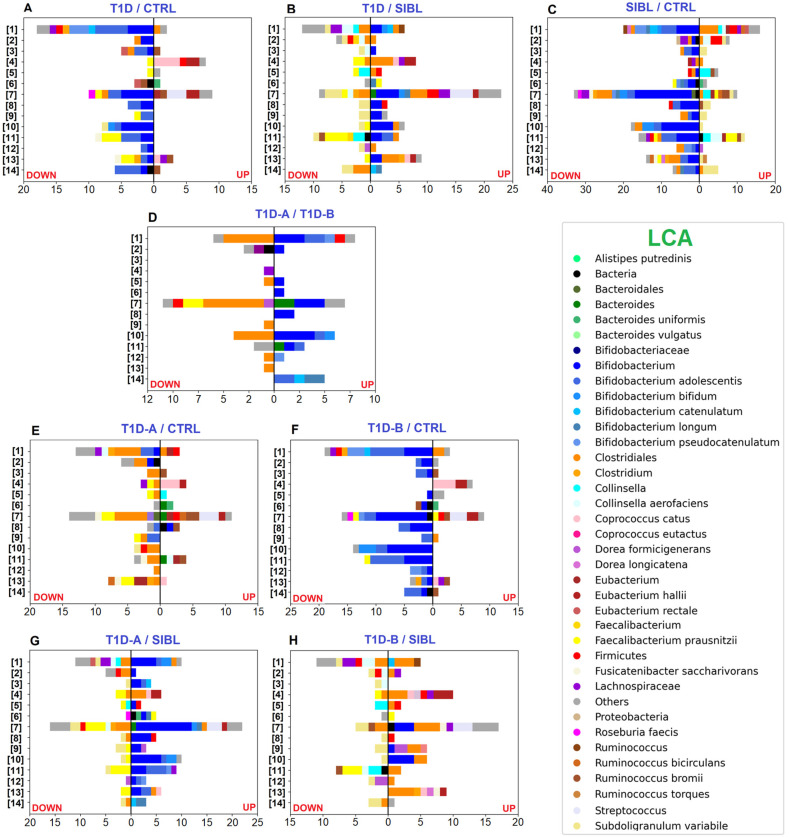
Bar charts plotting the number of statistically significant differential PGs, determined by the abundance ratio values in the comparison between groups of individuals and associated with functional annotation. Color code identifies taxa assigned to UP- (right) and DOWN-regulated (left) PGs by LCA. Selected pathways from the KEGG DB: [1] ABC transporters—[2] Alanine, aspartate, and glutamate metabolism—[3] Aminoacyl-tRNA biosynthesis—[4] Fatty acid degradation—[5] Fructose and mannose metabolism—[6] Galactose metabolism—[7] Glycolysis/Gluconeogenesis—[8] Oxidative Phosphorylation—[9] Pentose and glucoronate interconversion—[10] Pentose phosphate pathway—[11] Purine metabolism—[12] Pyrimidine metabolism—[13] Pyruvate metabolism—[14] Starch and sucrose metabolism. **Panels** (**A**–**C**): comparisons between T1D, CTRL, and SIBL groups. **Panel** (**D**): comparison between groups of patients stratified by severity (T1D-A and T1D-B). **Panels** (**E**,**F**): tests comparing T1D-A and T1D-B groups versus CTRL. **Panels** (**G**,**H**): tests comparing T1D-A and T1D-B groups versus SIBL.

Label-free quantitative (LFQ) analysis yielded a good number of statistically significant results with 223, 299, and 480 differentially regulated PGs by comparing T1D versus CTRL, T1D versus SIBL, and SIBL versus CTRL, respectively (Figure S1 and File S1). Among the 72 PGs up-regulated in T1Ds when compared with healthy CTRL, 24 were assigned to the *Bacteroides* genus or related species by the lowest common ancestor (LCA) algorithm and associated to functions belonging to the COG categories M (cell wall/membrane/envelope biogenesis), U (intracellular trafficking, secretion, and vesicular transport), and P (Inorganic ion transport and metabolism) (Figure 2A). Enhanced activity was also observed for *Eubacterium hallii* and *Coprococcus catus*, mostly related to COG category I (lipid transport and metabolism) and, more specifically, to fatty acid degradation (Figure 3A). Some metaproteins were linked to butyrate synthesis from acetyl-CoA, namely Acetyl-CoA C-acetyltranferase (COG0183), Enoyl-CoA hydratase (COG1024), 3-hydroxyacyl-CoA dehydrogenase (COG1250), and proteins from the Acyl-CoA dehydrogenase superfamily (COG1960), which also includes enzymes involved in amino acid catabolism (File S1) [33,34].

On the other hand, the majority of up-regulated PGs in CTRLs were associated with the *Bifidobacterium* genus with a strong prevalence of *Bifidobacterium adolescentis* when the LCA was assigned at the species level, mainly involved in functions enclosed in COG categories G (carbohydrate transport and metabolism), E (amino acid transport and metabolism), and C (energy production and conversion). Quite all the remaining PGs were assigned to the Clostridiales order or to *Faecalibacterium prausnitzii* at the species level with activities related to the COG categories C, E (amino acid transport and metabolism), and G. At a less broad functional annotation level, many dysregulated PGs were associated with the pentose phosphate pathway (PPP), glycolysis/gluconeogenesis, ABC transporters, galactose metabolism, and starch and sucrose metabolism, concerning Bifidobacteria, and with purine and pyruvate metabolism in the case of *F. prausnitzii* (Figure 3A).

Comparing T1Ds and SIBLs, 172 PGs were up-regulated in T1Ds and 127 more abundant in SIBLs, distributed between the same principal functions but apparently with different taxa accomplishing them (Figure 2B). In this case, Bifidobacteria activity was enhanced in T1Ds, predominantly related to COG category J (Translation, ribosomal structure, and biogenesis). Metaproteins belonging to COG categories C and G appeared instead associated with an increased activity of *Bifidobacterium* species and Clostridiales families in T1Ds, while *F. prausnitzii*, *Subdoligranulum variabile,* and *Collinsella* were augmented in SIBLs (File S1). In particular, *Collinsella* up-regulation seemed to be a peculiar feature in the SIBL group, almost replacing *Bifidobacterium* in the overall account of Actinobacteria. Although *Collinsella* has been put in relation to states of disease, such as in type 2 diabetes patients, but also positively correlated with circulating insulin in obese pregnant women, or proposed as probiotics for the treatment of inflammatory bowel disease, our results need further evidence to make any consideration [35]. More evident differences were observed in the KEGG pathway distribution, showing activities regarding ribosome, glycolysis/gluconeogenesis, PPP, and pyruvate metabolism enhanced in T1D and other functions such as ABC transport, purine metabolism, starch and sucrose metabolism, alanine, aspartate, and glutamate metabolism up-regulated in SIBLs (Figure 3B).

As in the case of T1Ds, a reduced level of metaproteins associated with Bifidobacteria was also observed in SIBLs when compared with CTRLs, counting 208 out of 315 statistically significant down-regulated PGs, while the others were mainly assigned to Firmicutes or, more specifically, to Clostridiales, Lachnospiraceae, *Ruminococcus*, *Fusicatenibacter saccharivorans*, and *Dorea longicatena*. Among the taxa associated with the 164 up-regulated PGs in SIBLs, Clostridiales were predominant and represented by *F. prausnitzii* and *S. variabile* as the most abundant species, followed by *Ruminococcus bromii*, *Ruminococcus bicirculans*, and *Dorea formicigenerans*. The remaining PGs were mainly associated with *Bacteroides* and *Collinsella* at the genus level (File S1). The principal functional trends were very similar to those observed comparing CTRLs with T1Ds, showing the main differences in the higher variability of taxa associated with up-regulated PGs, and in an apparent more balanced regulation of lipid metabolism (Figure 2C). The functional annotation based on the KEGG DB showed pyruvate metabolism also in this case increased in CTRLs together with the PPP, while purine metabolism, ABC transport, alanine, aspartate, and glutamate metabolism, and fructose and mannose metabolism were enhanced in SIBLs (Figure 3C).

### 2.3. GM Quantitative Analysis: T1D Patients’ Classification by Insulin Need

T1D patients were classified into two groups based on exogenous insulin need (IN) values as a T1D key clinical parameter, which is representative of pancreatic β-cell damage. From this point, the 14 T1D patients showing IN ≥1 IU/kg BM and the 25 patients showing IN < 1 IU/kg BM will be named T1D-A and T1D-B, respectively, all along the text. Further LFQ analyses were performed by comparing these subgroups to each other (T1D-A versus T1D-B) and also versus the CTRL and SIBL groups. A Pearson’s correlation test performed on the main clinical parameters showed a significant negative correlation of the IN with blood pH at the onset (*p*-value < 0.01), C-peptide, and anti-GAD antibodies (*p*-value < 0.05), while a positive correlation was observed with glycated hemoglobin (HbA1c, *p*-value < 0.001) (Figure S2). HbA1c and C-peptide measurements exceeded the threshold values for all 39 patients and for 36/39 patients, respectively; thus, these parameters could not be used to separate them into two comparable groups (Table 2).

Comparing the two T1D subgroups, 59 PGs were up-regulated in T1D-A, assigned quite exclusively to Bifidobacteria, while 74 PGs were up-regulated in T1D-B patients with the LCA set at the Clostridiales order level for the majority of cases (File S1). Functions annotated by COG categories showed G equally active in the two groups, while for E and C a higher number of up-regulated PGs were found for T1D-B (Figure 2D). Concerning the main pathways, glycolysis was more active in T1D-B, while starch and sucrose metabolism was associated only with up-regulated PGs in T1D-A (Figure 3D).

The impact of the severity degree of the disease was evaluated by stratifying the overall T1D group in T1D-A and T1D-B and comparing them with either CTRL or SIBL. In the tests versus CTRL, the up-regulation pattern in patients was similar for T1D-A and T1D-B, while T1D-B was shown to contribute more markedly to the overall down-regulation of the Bifidobacteria functions observed with the whole T1D group. Conversely, the lack of *F. prausnitzii* activity, mostly with regards to pyruvate metabolism, appeared to be ascribed to T1D-A (Figure 2E,F and Figure 3E,F). Similarly, the comparison of patients with SIBL showed that the up-regulation of PGs related to *Bifidobacterium* and the down-regulation of *F. prausnitzii* and *Subdoligranulum* activity was evident with T1D-A, while it was not with T1D-B. The increase in Clostridiales functionality and the down-regulation of *Collinsella* was instead observed more clearly with T1D-B (Figure 2G,H and Figure 3G,H).

### 2.4. Human Proteins

The protein extraction procedure from fecal samples was developed for bacteria enrichment so that only 5.8% of identifications were assigned to human sequences, deriving from epithelial cells and gut mucosa, and LFQ analysis yielded a very low number of differentially regulated human proteins (File S2). Some of these proteins, such as Chymotrypsin-C, Alpha-amylase 1C, Alpha-amylase 2B, Calcium-activated chloride channel regulator 1 and Chymotrypsinogen B2, were mapped to the KEGG pancreatic secretion pathway and also reported as down-regulated in T1D in metaproteomics studies, more focused on the GM-host proteome relationship [28,29,30]. Although the dysregulation of such proteins could provide interesting information about endocrine dysfunction, the impairment of exocrine pancreas functionality in T1D, and on the still unclear correlation with autoimmune β-cell destruction, our limited outcome could not be used to draw any robust considerations [36,37].

## 3. Discussion

Among the most relevant results of our investigation, we observed an up-regulation of *Bacteroides* activity, associated with a down-regulation of many PGs and functions related to *Bifidobacterium*, for T1D compared with CTRL, and both trends are also in good agreement with what has already been reported in recent metagenomic and metaproteomic studies [30,38,39,40]. Patient stratification by IN interestingly revealed that such divergent dysregulation was evident for T1D-B only when compared with CTRL, while a reduced *Bifidobacterium* activity was definitely not observed for T1D-A (Figure 2E,F and Figure 3E,F). Accordingly, in comparison with CTRL, T1D-A apparently contributed more than T1D-B to the *Bifidobacterium* increase with respect to SIBL, while in both groups a more similar distribution of taxa accomplishing down-regulated functions was observed (Figure 2G,H and Figure 3G,H). Thus, T1D-B patients, which should be associated with an earlier stage of the disease onset, showed this defect in *Bifidobacterium* functions, somehow similar to healthy SIBL. Consistent with what we observed, Patterson et al. reported a sudden increase of *Bacteroides* in streptozotocin-induced T1D mice, while *Bifidobacterium* increased after four weeks, when the disease could not be considered at an early stage [41]. If we assume that a higher IN actually represents a condition where T1D is already at a more advanced stage from the onset, *Bifidobacterium* reduced activity could be associated with the early triggering of the autoimmune response, possibly shared in the familiar environment in some cases, and considered as *primum movens* of the disease.

Microbial species involved in butyrate production are considered very important for host health due to their fundamental role in furnishing energy to cells from the colon epithelium and in regulating intestinal permeability [34]. In this sense, among the *Bifidobacterium* metaproteins and relative functions that we observed as down-regulated in T1D, the most likely responsible for a push toward an inflammatory process were those related to pathways involving short chain fatty acid (SCFA) synthesis, such as carbohydrate metabolism and energy production. Although Bifidobacteria are unable to directly synthetize butyrate, they can contribute to its production in a cross-feeding interaction with other bacteria that can metabolize lactate and acetate [42]. Bifidobacteria can use the PPP as an alternative glycolytic process to produce lactic and acetic acid from fructose-6-phosphate and indeed PGs involved in the PPP were down-regulated in T1D, such as Phosphogluconate dehydratase (COG0129), Transaldolase (COG0176), Transketolase (COG0021), and Phosphoketolase (COG3957). Pentose to glucoronate interconversion and oxidative phosphorylation were other down-regulated functions that would suggest reduced SCFA production and be related to a pro-inflammatory state [43]. On the other hand, T1D stratification by pancreatic β-cells damage showed that in patients with a higher IN, PGs associated with *Bifidobacterium* were not down-regulated when compared with CTRL and even up-regulated in the comparison with SIBL. Therefore, it remains difficult to draw definitive conclusions about the meaning of this marked *Bifidobacterium* decrement in T1D-B. This probably happens in the first weeks after diagnosis or even before the onset, since we observed it as well in their relatives. Bifidobacteria reduced activity would affect SCFA production and consequently contribute to an early inflammatory process. Alternatively, this down-regulation could be a consequence of another disequilibrium in the GM due to still not unveiled mechanisms.

Thus, from the perspective of a therapeutic intervention with probiotics on risky subjects, the use of Bifidobacteria, which was recognized to induce increased levels of insulin receptor proteins, could be promising to prevent or slow down the onset of T1D [44].

PGs assigned to *F. prausnitzii*, which is a direct butyrate producer, were decreased in T1D when compared with CTRL, mainly associated with purine and pyruvate metabolisms (Figure 3A) [45,46]. T1D patients’ classification by β-cells damage showed a defined picture where *F. prausnitzii* and other unspecified Clostridiales were associated with all the down-regulated PGs in T1D-A versus CTRL (Figure 2D and Figure 3D). It could also be conceivable that other down-regulated metaproteins could have been assigned to *F. prausnitzii,* but no taxon-specific peptides were detected due to highly conserved genes in the Clostridiales order, hampering the LCA algorithm to reach a more specific level.

**Figure 4:**
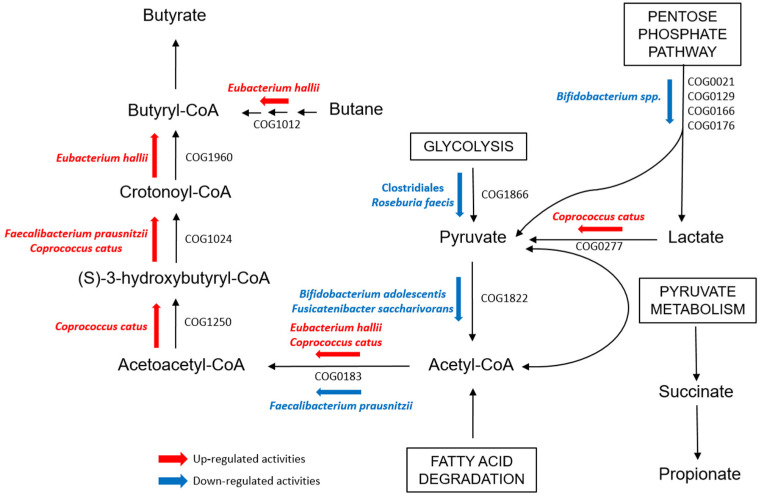
Scheme evidencing the detected up- and down-regulated bacterial functions involved in the butyrate pathway through Acetyl-CoA in the GM of T1D children when compared with that of healthy CTRL.

Other Clostridiales were instead observed to be more active in functions concerning SCFA metabolism in T1Ds. Fatty acid degradation, which is one of the pathways toward butyrate production, was up-regulated in both IN≥1- and IN<1-T1Ds compared to CTRLs. Particularly, PGs assigned to *C. catus* and *E. hallii* were associated with COGs involved in the pathway that brings to butyrate synthesis from acetyl-CoA, namely Acetyl-CoA C-acetyltranferase (COG0183), Enoyl-CoA hydratase (COG1024), 3-hydroxyacyl-CoA dehydrogenase (COG1250), and proteins from the Acyl-CoA dehydrogenase superfamily (COG1960), which also includes enzymes involved in amino acids catabolism (Figure 4) [33,34]. Our results could be enforced by an integration with metabolomic data to get a useful snapshot of bacterial metabolite abundance and better interpret our functional outcomes. Indeed, the role of SCFA metabolism in T1D onset, particularly regarding the mechanisms related to autoimmunity, has yet to be defined, as no definitive results raised in this work or have been reported in previous studies [47,48].

*Bacteroides* and related activities were observed up-regulated in both T1D-A and T1D-B versus CTRLs, finding in accordance with some studies but also in contrast with others, revealing an opposite trend in T1D patients from neighboring countries, suggesting a dependence of the *Bacteroides* abundance on environmental factors [16,18,49,50,51]. Regarding dietary habits, the cohort studied in the present work was composed of subjects living in Italy and following a generic Mediterranean diet. Moreover, given that all samples from patients in this study were collected at the onset of the disease, specifically at the moment of hospital admittance, no consequence of a particular diet for diabetes-affected individuals was taken into consideration to evaluate the GM profiles. Among the up-regulated metaproteins belonging to COG categories M and P, opacity proteins (COG3637), porins and outer membrane proteins were present (COG1538, COG4206, COG4774), being known for their immunogenic role and confirming that such enhanced activity by *Bacteroides* seemed somehow linked to pathogenesis in this particular case [52,53]. Many outer membrane proteins were involved in iron uptake and transport (COG4771, COG4772, COG1629), intriguingly suggesting a possible cause for high levels of iron in host tissues, a feature known to be correlated with insulin resistance risk [54]. Worthy of consideration were also the biopolymer transporters ExbD, ExbB/ToIQ (COG0848, COG0811), associated with the COG category U, strongly related with the TonB system, and recently raising interest about other mechanisms of bacterial pathogenicity [52]. Unlike Bifidobacteria, *Bacteroides* functions seem to be related to inflammation through more than one pathway; thus, it can be supposed to contribute to the disease onset. Nevertheless, its up-regulation was observed in patients regardless of IN, suggesting enhanced activity at a later stage of the disease, resulting in a more definite microbial signature for T1D.

## 4. Materials and Methods

### 4.1. Subjects Enrollment and Sample Collection

A cohort of pediatric patients with T1D at the onset, and some of their unaffected SIBL, was recruited between April 2017 and July 2019 at the Diabetes & Growth Disorders Unit of Bambino Gesù Children’s Hospital (OPBG) in Rome, Italy (Table 1). Fecal samples were collected from 39 T1D children (aged 4–15 years), 15 SIBL (aged 5–18 years), and 30 healthy children (CTRL, aged 4–17 years), from the Biobanking and Biomolecular Resources Research Infrastructure of Italy (BBMRI), European Research Infrastructure Consortium for Human Microbiome of the OPBG Human Microbiome Unit, were collected and stored at −80 °C, until further meta-omic processing. T1D patients included in the study must show glycemia values of >126 mg/dL, glycated hemoglobin (HbA1c)>6.5% (48 mmol/mL), c-peptide < 1 ng/mL among clinical parameters recorded at hospital admission (Table 2). All subjects with chronic diseases, those who had taken antibiotics, prebiotics, or probiotics in the previous two months were excluded from recruiting. The same criteria were applied in the CTRL group selection. In addition, the absence of gastro intestinal (GI) disorders and familiarity with autoimmune diseases was checked.

The study protocol was performed in accordance with the Principles of Good Clinical Practice and Declaration of Helsinki and was approved by the OPBG Ethics Committee (protocol number 1274_OPBG_2016). Written informed consent from either parents or legal representatives of children was obtained for participation in the study.

### 4.2. Protein Extraction and Digestion

Three-hundred mg of thawed fecal material was suspended in 6 mL of ice-cold phosphate buffer (DPBS; 200 mg/L KCl, 200 mg/L KH_2_PO_4_, 8000 mg/L NaCl, 1150 mg/L Na_2_HPO_4_) with thorough vortexing, then shaken 10 min, and the slurry was centrifuged 15 min at 402× *g* and 4 °C. The supernatant was collected, and the above procedure was repeated twice on the pellet. All the resulting supernatants for each sample were combined and centrifuged two more times at 402× *g* for 15 min, 4 °C, to remove debris. The supernatant was then centrifuged at 16,000× g, for 15 min, 4 °C, to obtain the cell pellet, which was resuspended in ice-cold DPBS and washed three times. The final bacterial cell pellet was resuspended in 300 μL of lysis buffer [4% sodium dodecyl sulfate (SDS), 100 mM Dithiothreitol (DTT) in 50 mM Tris-HCl pH 8, with addition of Halt Protease and Phosphatase Inhibitor Cocktail (Thermo Fisher Scientific, Waltham, MA, USA)], incubated at 95 °C for 15 min in agitation, then sonicated using a probe (VibraCell Ultrasonic Liquid Processor, Sonics & Materials Inc, Newtown, CT, USA), applying 25% amplitude for 3 cycle of 30 s. Through high-speed centrifugation at 16,000× *g* at room temperature for 10 min, the final supernatant, the protein extract, was collected and subjected to protein dosage using a 2-D Quant kit (GE Healtcare, Chicago, IL, USA).

Fifty μg of each protein extract was then reduced, alkylated, and digested for the subsequent mass spectrometry (MS) analysis by the filter-aided sample preparation (FASP) method, as already described elsewhere [55]. Enzymatic digestion was carried out overnight at 37 °C with Sequencing grade Trypsin (Promega, Milan, Italy) at a 1:50 ratio of enzyme to sample. After elution from the Microcon-10kDa Centrifugal Filter Unit (Merck, Billerica, MA, USA), the enzymatic reaction was quenched by adding trifluoroacetic acid (TFA) at a final concentration of 1%, and the solution was speedvac-dried. Recovered peptides were suspended in 2% acetonitrile (ACN), 0.1% formic acid (FA), and 97.9% water, and their quantity was assessed in comparison with a standard curve of MassPrep Escherichia coli digestion (Waters, Milford, Massachusetts, USA) by spectrophotometer measures (NanoDrop 2000, Thermo Fisher Scientific).

### 4.3. Chromatography and Mass Spectrometry Analysis

NanoLiquid Chromatography-ElectroSpray Ionization-tandem mass spectrometry (nLC-ESI-MS/MS) experiments were performed on an UltiMate3000 RSLCnano System directly coupled to an Orbitrap Fusion Tribrid mass spectrometer with a nanoESI source (EASY-Spray NG) (Thermo Fisher Scientific, Waltham, MA, USA). Injected tryptic peptides (1.95 μg) were at first trapped onto a μ-Precolumn cartridge C18 PepMap100 (5 µm particle size, 100 Å pore size, 300 µm i.d. x 5 mm length, Thermo Fisher Scientific) for desalting and focusing, using a flow rate of 10 μL/min for 3 min with an aqueous mobile phase (MP) with 2% ACN and 0.1% TFA. Gradient elution was carried out using an EASY-Spray PepMap RSLC C18 column (2 μm particle size, 100 Å pore size, 75 μm i.d., 50 cm length, Thermo Fisher Scientific) kept at 35 °C, with a flow rate of 250 nL/min and applying a one-step linear gradient starting from 95% MP-A (0.1% FA in water) and arriving at 25% MP-B (99.9% ACN, 0.1% FA) in 113 min, and total LC-run of 160 min. The Orbitrap analyzer was used for precursor ion detection (MS1) at resolving powers of 120 K (at 200 *m*/*z*), while fragment ions (MS2) were analyzed using the Ion Trap with a rapid scan rate. Data dependent analysis (DDA) was performed in top speed mode with a 3 s cycle-time, during which the most abundant multiple-charged (2^+^–7^+^) precursor ions were detected in the mass range of 375−1500 m/z. Quadrupole isolation with a 1.6 m/z isolation window was used, and dynamic exclusion was enabled for 60 s after 2 scans. High-energy collisional dissociation (HCD) was applied for fragmentation using a 30% normalized collision energy. Automatic gain control targets were set to 4.0 × 10^5^ for MS1 and 2 × 10^3^ for MS2, using 50 and 300 ms as maximum injection times, respectively. The signal intensity threshold for MS2 was set to 5 × 10^3^, and the option “Injection Ions for All Available Parallelizable Time” was enabled. For internal calibration, a lock mass was set choosing the polydimethylcyclosiloxane signal (445.12003 *m*/*z*) that was constantly detected during acquisition. Two technical replicates were recorded for each sample.

### 4.4. Bioinformatic Data Processing and Metaproteomic Analysis

nLC-ESI-MS/MS raw data were processed and analyzed by the MetaLab 1.2 desktop version working with MaxQuant version 1.6.5 [56,57]. Search parameters were set as follows: carbamidomethylation of cysteine as fixed modification, protein N-term acetylation and oxidation of methionine as variable modification, high-low instrument resolution. Databank search was performed versus the IGC—“Integrated reference catalog of the human gut microbiome”, Human Gut (http://meta.genomics.cn/—9.9M) and Homo sapiens UniProtKB/Swiss-Prot (version 2020_01—26 February 2020) DB. The specific DB creation in MetaLab was performed by selecting the spectra clustering strategy.

MetaLab yielded a PG list that was reduced through progressive filtering operations. In the first step, proteins are filtered by selecting the ones that have a number of detected peptides ≥2 with at least a single one defined as unique for the PG. Peptides and protein intensities were averaged from the results of the two technical replicates, and then protein matches obtained against the bacterial and human DBs were split and treated separately for the subsequent processing steps. The PG intensities for each individual were determined by averaging the intensities of all the proteins being part of the same PG. A log10 transformation was applied to the LFQ intensities of the PGs, and only those PGs with valid LFQ intensity values in ≥50% of the whole sample set were used for statistical analysis. Finally, all missing values were imputed by a KNN imputer using a neighborhood of $\;\sqrt{\mathrm{patients}}$. The resulting filtered matrices were merged together to retrieve a single comprehensive table where taxonomy (LCA rank and the taxon assignment at all levels), functional annotation (COG ID, COG name, COG category, NOG name, KEGG ID, KEGG name, and GO), and LFQ intensities were associated with each PG. All the processing steps and the subsequent statistical analyses were automated by means of ad hoc Python 3.7 scripts (Figure S3). The main packages used are pandas, numpy, scipy, and scikit-learn.

Functional annotation for detected human proteins was carried out using the iProXpress (integrated Protein eXpression) web application (https://proteininformationresource.org/iproxpress2/) accessed on February 2022.

### 4.5. Statistics

Alpha- and beta-diversity were computed by skbio-diversity using analysis of variance (ANOVA test) and permutational analysis of variance (PERMANOVA test), respectively; the latter was applied to Bray–Curtis and Euclidean distance matrices. The relative LFQ analysis was performed using a *t*-test to compare the LFQ intensities in couples of sample groups. Significant values for the fold change (FC) were considered to be those with abundance ratio ≥1.5 and ≤0.66, and *p*-value < 0.05. Volcano plots were displayed using log10 [abundance ratio] and −log10 [*p*-value] (Figure S2). The adjusted p-value for false discovery rate (FDR) was computed by applying the Benjamini/Hochberg correction for independent or positively correlated tests. We also computed the effect size of the *t*-tests using Cohen’s d, which quantifies the magnitude of effect present in the population.

## 5. Conclusions

In this study, a cohort of T1D children at the onset was studied and compared with an age-matched cohort of healthy CTRLs and a smaller group of siblings to individuate specific gut microbial features, possibly contributing to the insurgence of the disease. In a context where the number of reported metaproteomic investigations on this topic is rather scarce, our work is probably the first to provide a comprehensive outcome where the relative abundance of taxa was put in relation to the functions they accomplish in the GM. Given the accordance with many previously reported observations by 16S rRNA metagenomics, our results allowed us to identify some microbial patterns associated with the disease onset and actually involving functional pathways related to inflammation and immune response. We showed evidence of an up-regulation of *Bacteroides* functions related to the immune response in the whole group of T1D children, compared with CTRL, evidencing GM features linked to T1D pathogenesis. Down-regulation was instead observed for *F. prausnitzii*, suggesting a possible alteration of the SCFA metabolism and a consequent relation with inflammation. More interesting was the reduced *Bifidobacterium* activity that was mainly observed in those patients where the β-cell damage was supposed to be lower and also in the smaller group of healthy siblings. According to other observations, it seems possible that such a characteristic of the GM, which can be associated more with a state prior to the onset, rather than with a situation of overt disease.

## Figures and Tables

**Table 1:** Study cohort’s characteristics.

	CTRL	T1D	SIBL
Mean age in years (± SD ^1^)	10 ± 3.2	10 ± 3.2	13 ± 4.4
Gender (male/female)	14/16	22/17	6/9

^1^ SD: standard deviation.

**Table 2:** Clinical parameters recorded for T1D patients at hospital admission and respective reference values for T1D diagnosis.

Clinical Parameters	Average	Standard Deviation	Reference Value	Patients with Values Exceeding the Reference Value
Age (years)	9.56	3.19	≥14	4/39
IAA (U/mL)	7.56	4.76	≥7	16/34
IA2 (U/mL)	11.78	12.79	≥1	32/37
anti GAD (U/mL)	36.79	45.39	≥1	28/37
BMI (kg/m^2^)	16.44	2.95	≤14	10/39
Blood pH at onset	7.27	0.11	≤7.3	19/39
Exogenous insulin need (IU/kg BM)	0.87	0.28	≥1	14/39
C-peptide (ng/mL)	0.29	0.19	<0.6	36/39
HbA1c (%)	101.13	23.46	>38	39/39
CRP (mg/L)	0.56	1.61	>1	4/39

BMI, body mass index; IAA, insulin autoantibodies; IA2, islet antigen 2 antibody; anti-GAD, anti-glutamic acid decarboxylase antibody; HbA1c, glycated haemoglobin; CRP, C-Reactive Protein.

## Data Availability

All recorded mass spectra can be retrieved at the MASSIVE repository with the identifier code MSV000089691.

## Supplementary Materials

The following supporting information can be downloaded at: https://www.mdpi.com/article/10.3390/ijms232415982/s1, Figure S1: Volcano plots showing significant metaprotein dysregulation in all the discussed t-tests after label-free quantitative analysis; Figure S2: Correlation heatmap of clinical characteristics based on the Pearson’s correlation metric; Figure S3: Scheme of the bioinformatic automated workflow for the metaproteomic analysis; File S1: Excel worksheets resuming the results of the LFQ analysis performed on bacterial proteins; File S2: Excel worksheets resuming the results of the LFQ analysis performed on human proteins
