# Supplementary material for: Functional and Taxonomic Traits of the Gut Microbiota in Type 1 Diabetes Children at the Onset: A Metaproteomic Study
Source: Int J Mol Sci. 2022 Dec 15;23(24):15982. doi: 10.3390/ijms232415982 (PMC9787575; doi:10.3390/ijms232415982)
Supplement: Supplementary file 1 [file ijms-23-15982-s001.zip › Supplementary Figures.pdf]

Supplementary Material

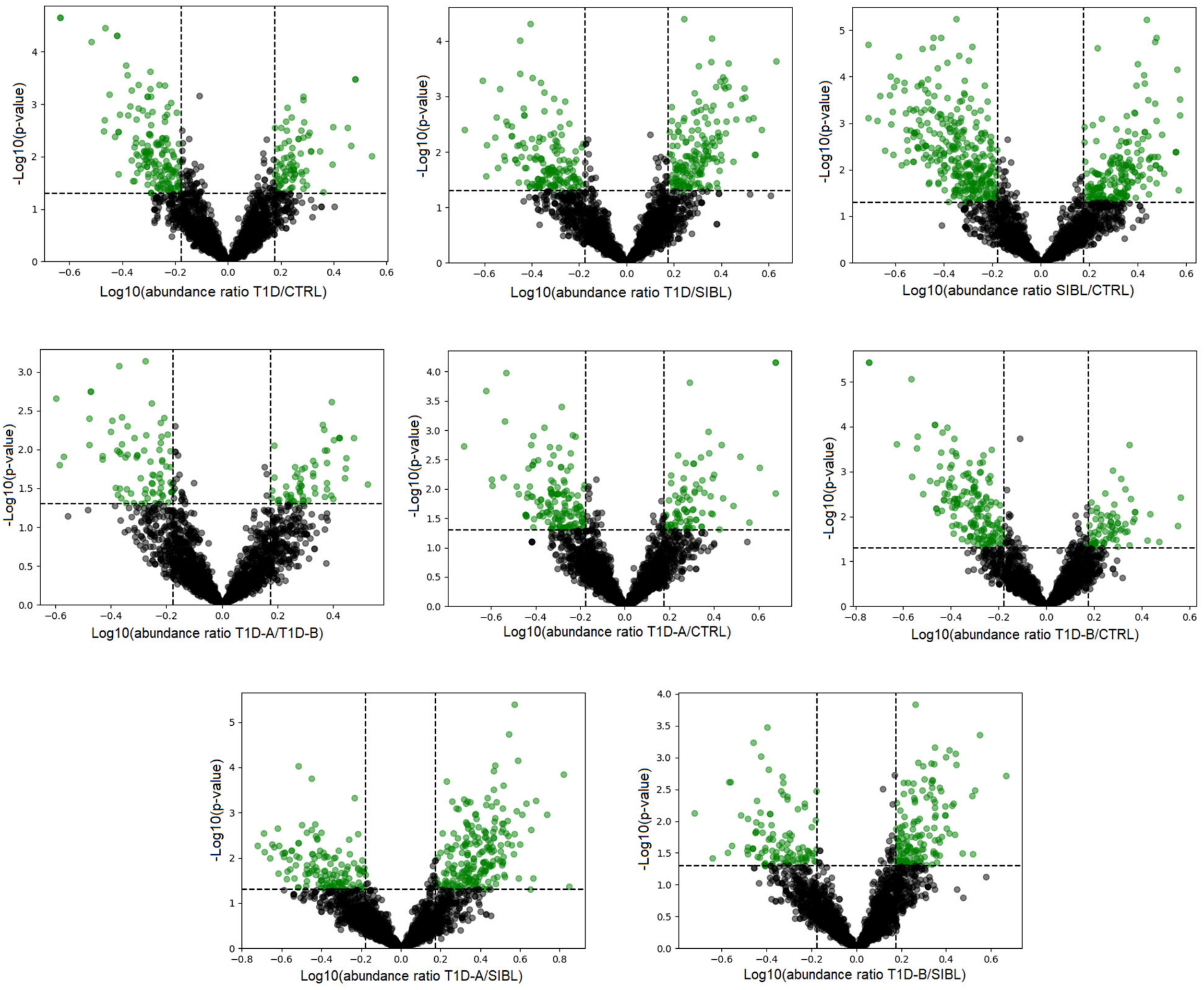

Figure S1. Volcano plots showing significant metaprotein dysregulation in all the discussed t-tests after label-free quantitative analysis. Significant values (spots marked in green) were selected by setting a p-value threshold <0.05 and considering abundance ratios >1.5 and <0.66.

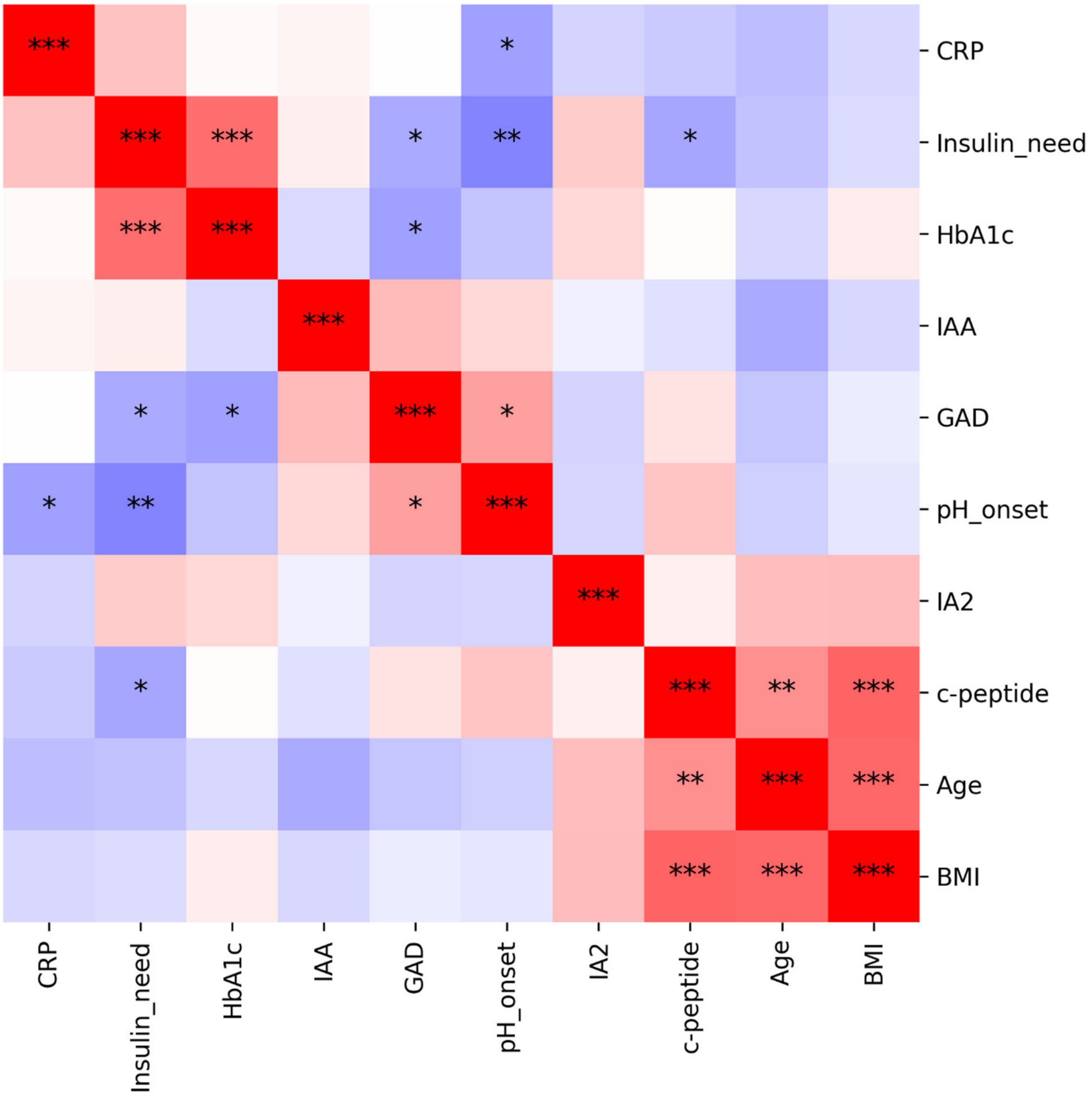

Figure S2. Correlation heatmap of clinical characteristics based on the Pearson's correlation metric.

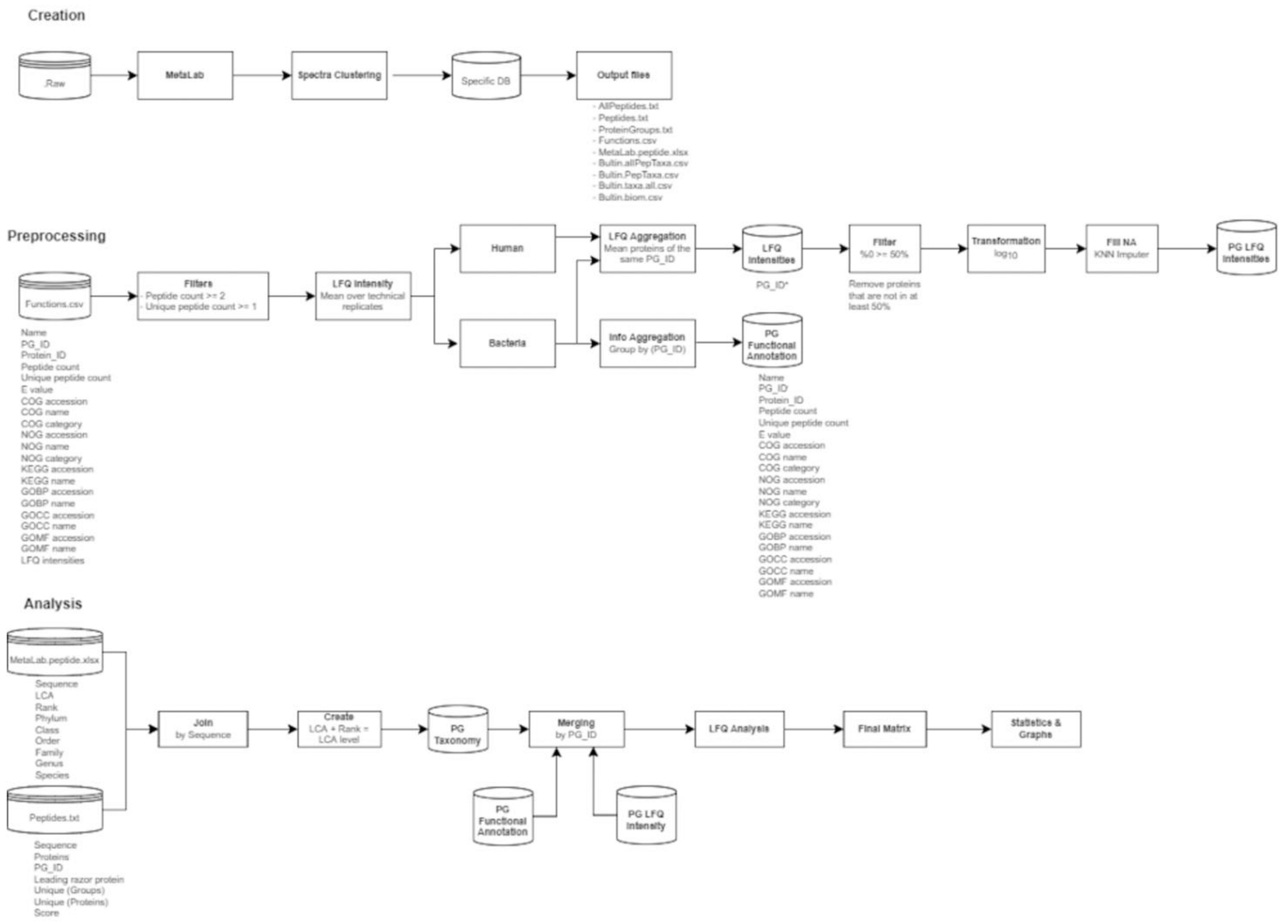

Figure S3. Scheme of the bioinformatic automated workflow for the metaproteomic analysis. Raw data were uploaded in MetaLab and the resulting output files were managed by an ad-hoc python script to obtain a single comprehensive matrix. Peptides and protein intensities were averaged from the results of the two technical replicates, and PG intensities for each individual were determined by averaging the intensities of all the proteins being part of the same PG. A log10 transformation was applied to the label-free quantitative (LFQ) intensities of the PGs and only those PGs with valid LFQ intensity values in ≥ 50% of the whole sample set were used for statistical analysis. Finally, all missing values were imputed by a KNN imputer using a neighborhood of $\sqrt{patients}$. The resulting filtered matrices were merged together to retrieve a single comprehensive table where taxonomy (LCA rank and the taxon assignment at all levels), functional annotations and LFQ intensities were associated with each PG.
